# OCT4 Positively Regulates Survivin Expression to Promote Cancer Cell Proliferation and Leads to Poor Prognosis in Esophageal Squamous Cell Carcinoma

**DOI:** 10.1371/journal.pone.0049693

**Published:** 2012-11-21

**Authors:** Chunguang Li, Yan Yan, Weidan Ji, Longlong Bao, Haihua Qian, Lei Chen, Mengchao Wu, Hezhong Chen, Zhigang Li, Changqing Su

**Affiliations:** 1 Department of Molecular Oncology, Eastern Hepatobiliary Surgical Hospital, Second Military Medical University, Shanghai, People’s Republic of China; 2 Department of Cardiothoracic Surgery, Changhai Hospital, Second Military Medical University, Shanghai, People’s Republic of China; Peter MacCallum Cancer Centre, Australia

## Abstract

**Background:**

OCT4 and Survivin are important factors for cancer cell proliferation, renewal and dedifferentiation, and correlate with resistance to radiotherapy and chemotherapy in most human cancers, but their regulatory mechanisms are not well known.

**Methodology/Principal Findings:**

In this study, 50 patients with esophageal squamous cell carcinoma (ESCC) were retrospectively analyzed. OCT4 was expressed in 13 cases (26%), and survivin was positively expressed in 31 cases (62%), examined by immunochemistry. OCT4 was found to be an independent predictive factor for median survival time, and the patients from the subgroup with both high expression of OCT4 and Survivin had the worst prognosis investigated by log-rank test. To further explore the molecular regulatory mechanism between OCT4 and Survivin, we constructed the specific *small* hairpin RNA (shRNA)-expressing vectors targeting OCT4 or/and Survivin and manipulated the expression of OCT4 and Survivin. By Western blotting and RT-PCR, we found that OCT4 could up-regulate Survivin expression in the esophageal cancer cell lines Eca109 and TE1. Simultaneously knockdown of OCT4 and Survivin expression induced cell apoptosis and G2-phase decrease of cell cycle by flow cytometry, and finally exerted an enhanced anti-proliferation potency in Eca109 and TE1 cell lines by MTT assay.

**Conclusions:**

This study shows that OCT4 and Survivin expression were correlated with poor survival in patients with ESCC. OCT4 and Survivin may be regarded as targets in ESCC biotherapy.

## Introduction

Esophageal squamous cell carcinoma (ESCC) is one of most malignant tumors with high mortality [1], [2]. Although some new molecular targets have been found and used in ESCC biotherapy, the molecular mechanisms of ESCC recurrence and metastasis are still not understood. A growing body of evidence suggested that only a small fraction of cancer initiating cells have the ability to self-renew, as well as, to drive initiation and progression of cancer, and presented strongly resistance to chemotherapy and radiotherapy [3], [4], which gives us a better understanding of molecular basis of ESCC.

Octamer-binding transcription factor 4 (OCT4) is one of the stem related transcription factors, regulating tumor proliferation and self-renewal. Poorly differentiated or undifferentiated cancer cells have been characterized by many phenotypic traits similar to undifferentiated embryonic stem cells, suggesting that OCT4 may be expressed in solid tumors as a cancer initiating cell biomarker [5]. OCT4 belongs to the family of POU-domain transcription factors, including a homeodomain which is important in embryonic development [6], [7]. It has been proved that OCT4 overexpressed in a lot of somatic cancers, such as breast cancer, prostate cancer, non-small cell lung cancer, bladder cancer, oral squamous cell carcinoma, gastric cancer, esophageal cancer [8]–[14]. OCT4 expression plays a pivotal link in tumorigenesis and maintainance of cancer cells.

Survivin is a member of the inhibitors of the apoptotic gene family, and plays an important role in tumor progression by inhibiting cell apoptosis, regulation of cell division, and induction of angiogenesis [15]. Overexpression of Survivin was proposed in various cancers including ESCC [16], [17], but rarely present in normal adult tissues. Survivin expression in circulating cancer cells in the peripheral blood of patients with ESCC was detected and provided valuable information in the prediction of cancer recurrence and poor prognosis [18], [19]. Besides, overexpression of Survivin in ESCC presented resistance to chemotherapy and shorter survival [20], and there were similar results in other cancers [21].

Previous study demonstrated that knockdown of Survivin expression in a number of human cancer cell lines, such as A549, HeLa and MCF-7 cells, resulted in a significant reduction of cell viability, and combination of Survivin-directed silencing strategy with chemotherapeutic agents constituted a valuable approach for cancer treatment with an enhanced antitumor efficacy [21], [22]. However, cancer re-growth is probably the most important feature, because the cancer initiating cells resist the conventional cancer therapies and are likely to play a major role in cancer relapse [23]. Therefore, targeting cancer initiating cells has the potential to significantly improve outcomes for cancer patients. OCT4 is a master gene that plays a key role in the self-renewal and pluripotency of stem cells. Being selectively expressed in tumor tissues, evidence suggested that OCT4 may be a promising target for development of anticancer strategies to eliminate cancer initiating cells [24].

Recently, it was reported that Survivin expression was dramatically decreased in OCT4 knockdown murine embryonic stem cells [25], suggesting that there is a relationship between OCT4 and Survivin. But the molecular regulatory mechanisms between OCT4 and Survivin are not yet clear in cancers. In the current study, we examined OCT4 and Survivin expression and analyzed the prognostic relevance of these two genes with ESCC specimens. Meanwhile, the regulatory mechanism of OCT4 and Survivin expression and their function on cell apoptosis, cell proliferation or cell cycle were investigated in ESCC cell lines.

## Results

### OCT4 and Survivin were Over-expressed in ESCC

**Figure 1:**
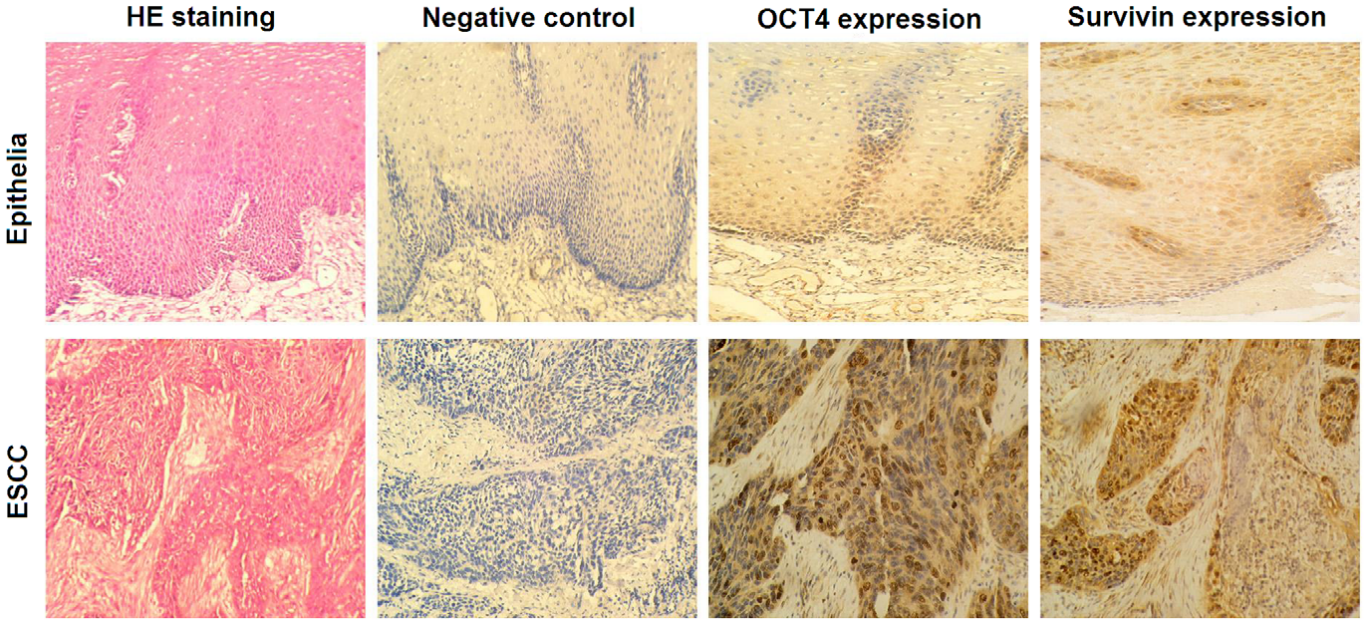
OCT4 and Survivin expression were detected by immunohistochemical staining in the resected specimens of esophageal squamous cell carcinoma (ESCC) and adjacent esophageal mucosa. The paraffin-embedded sections were subjected to immunohistochemical examination with the primary mouse anti-human OCT4 and rabbit anti-human Survivin antibodies at working concentration of 1∶100, and the diaminobenzidine (DAB) was used to stain the positive reaction. The phosphate buffered saline (PBS), instead of the primary antibodies, was used for negative control. Percentages of positive cells were counted within 5 high-power fields, original magnification ×200.

The expression of OCT4 and Survivin was detected by immunohistochemistry in the specimens of ESCC and adjacent normal esophageal tissues. OCT4 was expressed in 13 (26%) of ESCC but only 2 (4%) of normal esophageal tissues. Survivin was expressed in 31 (62%) of ESCC but only 11 (22%) of normal esophageal tissues. There were differences between ESCC and normal esophageal groups (*p* = 0.0051 for OCT4; *p* = 0.0001 for Survivin). The OCT4-positive immunoreactivity was mainly distributed in ESCC cellular nuclei and Survivin was mainly distributed in ESCC cytoplasm. The OCT4- and Survivin-positive cells were primarily located in the basal parts of the epithelia (Fig. 1). Survivin expression did not related with OCT4 expression in these ESCC samples (R = 0.276, *p* = 0.052; Table 1).

**Table 1:** Relationship between OCT4 and Survivin expression in ESCC samples.

OCT4	Survivin		
	Positive	Negative	Total
Positive	11	2	13
Negative	20	17	37
Total	31	19	50

R = 0.276, *p* = 0.052.

Statistical correlation between OCT4 or Survivin expression and ESCC clinicopathological characteristics (gender, age, cell differentiation, tumor invasion depth, lymph node metastasis) was analyzed and revealed no significant differences between the OCT4- or Survivin-positive and OCT4- or Survivin-negative cases of ESCC (Table 2).

**Table 2:** Correlation between OCT4 or Survivin expression and ESCC clinicopathological features.

Variables	n	OCT4Positive	OCT4 Negative	χ^2^	*p*	Survivin Positive	Survivin Negative	χ^2^	*p*
**Gender**				0.002	0.968			0.002	0.968
Male	37	23	14			23	14		
Female	13	8	5			8	5		
**Age**				0.071	0.790			0.071	0.790
≤60 y	24	15	8			15	8		
>60 y	26	16	10			16	10		
**Differentiation**				3.295	0.192			3.295	0.192
Grade 1	14	6	8			6	8		
Grade 2	31	21	10			21	10		
Grade 3	5	4	1			4	1		
**p-T**				1.438	0.230			1.438	0.230
T1/2	16	8	8			8	8		
T3	34	23	11			23	11		
**p-N**				0.002	0.968			0.002	0.968
N_0_	13	8	5			8	5		
N1/2	37	23	14			23	14		

### OCT4 and Survivin Correlated to Poor Prognosis of ESCC Patients

**Figure 2:**
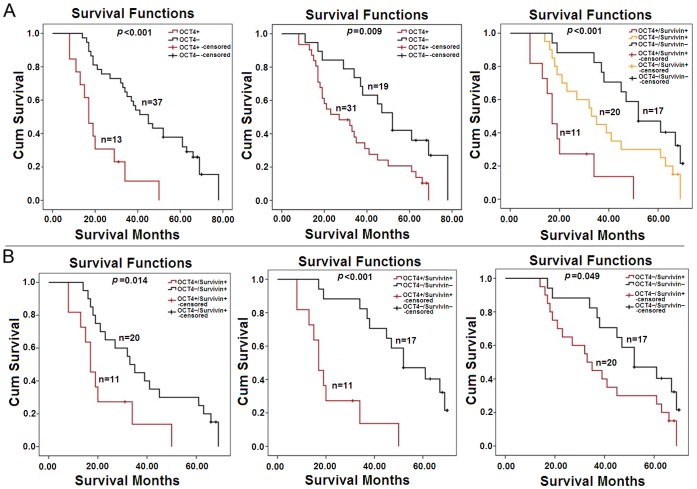
Comparison of overall survival (OS) curves for 50 patients underwent the radical esophagectomy of ESCC. (A) Fifty patients with ESCC were enrolled in the study, and the OS was defined as the time period from the operation date to the date of death or the end date of follow-up. Survival curves were calculated by the Kaplan-Meier method, and the significant difference was tested using log-rank test. (B) Every two subgroups were compared by log-rank test.

Follow-up data of 50 patients were analyzed using the Kaplan-Meier method to estimate survival curves. The median OS was 34.5 months. The median survival of ESCC patients with OCT4 positive expression was significantly less than that of patients with OCT4 negative expression (*p*<0.001). The similar result was showed between Survivin-positive and -negative ESCC cases (*p* = 0.009). Among the three subgroups (OCT4-positive/Survivin-positive, OCT4-negative/Survivin-positive, OCT4-negative/Survivin-negative), patients with OCT4-positive/Survivin-positive ESCC had a significantly poorest prognosis (*p*<0.001), and the longest OS was documented in OCT4-negative/Survivin-negative subgroup (Fig. 2A). Further analysis was performed between any two subgroups by log-rank test, and the results showed that OCT4 and Survivin expression were strongly associated with poor prognosis of ESCC patients (Fig. 2B).

Data analysis with the univariate Cox’s proportional hazard model revealed that OCT4 and Survivin were significant prognostic factors of ESCC patients. However, the multivariate analysis showed that OCT4 was an independent prognostic value in ESCC patients, but Survivin was not (*p* = 0.168; Table 3).

**Table 3:** Univariate and Multivariate Cox’s propotional hazard models (n = 50).

Term	Risk ratio	95% confidence interval	*p* value
**Univariate**			
Grade	1.289	0.761–2.184	0.345
p-T	1.256	0.780–2.024	0.384
p-N	0.921	0.448–1.894	0.823
OCT4	4.233	2.011–8.910	0.000
Survivin	2.352	1.201–4.606	0.013
**Multivariate**			
p- Grade	1.313	0.727–2.371	0.367
p-T	1.225	0.720–2.083	0.454
p-N	0.988	0.437–2.233	0.977
OCT4	3.670	1.598–8.431	0.002
Survivin	1.694	0.801–3.584	0.168

### Inhibitory Effect of shRNA Vectors Targeting OCT4 and Survivin in ESCC Cell Lines

**Figure 3:**
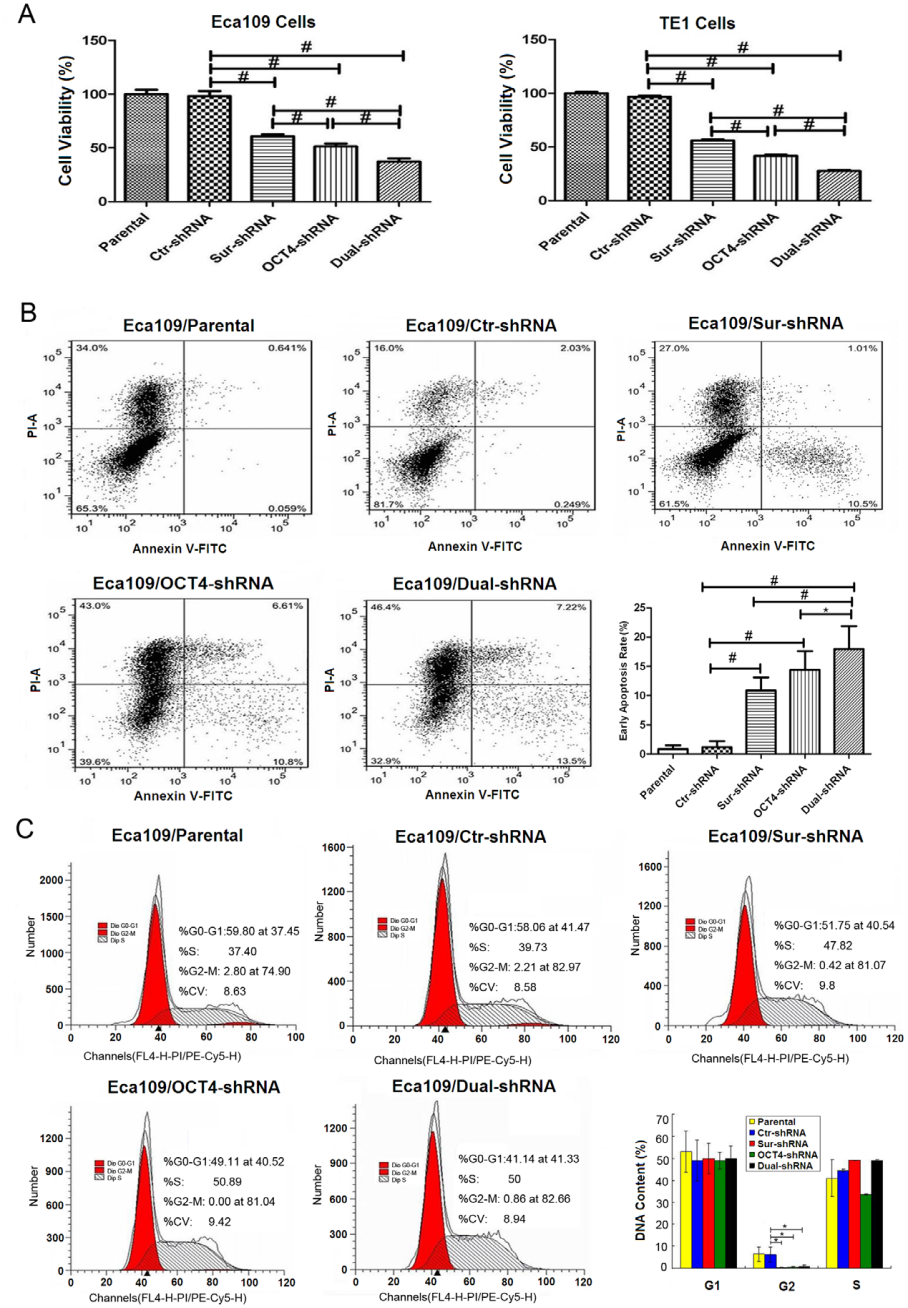
Co-Suppression of OCT4 and Survivin effectively inhibited tumor cell growth. (A) The parental and shRNA-transfected Eca109 and TE1 cells were cultured in 96-well plates at a density of 8×10^3^ cells/well for 48 h. Cell viability was determined by methylthiazoletetrazolium (MTT) assay at a wavelength of 490 nm, ^#^ *p*<0.01; Dual-shRNA: vector containing double shRNAs targeting both OCT4 and Survivin. (B) After Eca109 cells were transfected with shRNA vectors, 5×10^6^ cells/ml were harvested and stained with Annexin V-FITC and PI, and analyzed by flow cytometry. Percentages of early apoptosis were shown in histograms, data were representatives of three independent experiments, and quantitative histograms were pooled data, error bars are standard deviation (SD), *p<0.05; ^#^ *p*<0.01. (C) Cell cycle was measured by flow cytometry. Data of cell cycle were shown in histograms, *p<0.05.

To indentify whether the specific *small* hairpin RNA (shRNA) targeting OCT4 *(OCT4-shRNA),* Survivin *(Sur-shRNA)*, or double shRNAs (Dual-shRNA) targeting both OCT4 and Survivin, influenced esophageal cancer cell proliferation, MTT assay was performed to detect cell viability. Cell viability was obviously decreased in the Eca109 and TE1 cells transfected with OCT4-shRNA and Sur-shRNA when compared with the parental or Ctr-shRNA transfected cells. Dual-shRNA exerted an enhanced inhibitory effect on cell viability compared with the shRNA vector targeting the single factor. However, there was no difference in cell viability between OCT4-shRNA and Sur-shRNA groups (Fig. 3A).

Apoptosis of ESCC cell lines was measured by FCM with Anexin V-FITC and PI staining. After treatment with shRNA vectors for 48 h, the percentages of early cell apoptosis were increased in OCT4-shRNA (13.4±2.76%), Sur-shRNA (10.89±2.21%) and Dual-shRNA (17.79±3.89%) groups, compared with that in the parental cells (1.20±1.01%) and Ctr-shRNA (0.87±0.64%) group, especially higher in Dual-shRNA group (Fig. 3B). Compared with the parental and Ctr-shRNA groups, the percentages of G2-phase cells were significantly reduced in the OCT4-shRNA, Sur-shRNA and Dual-shRNA transfected groups, but there were no significantly differences for the G1 and S phase cells (Fig. 3C).

### Survivin Expression was Associated with OCT4 in ESCC Cells

**Figure 4:**
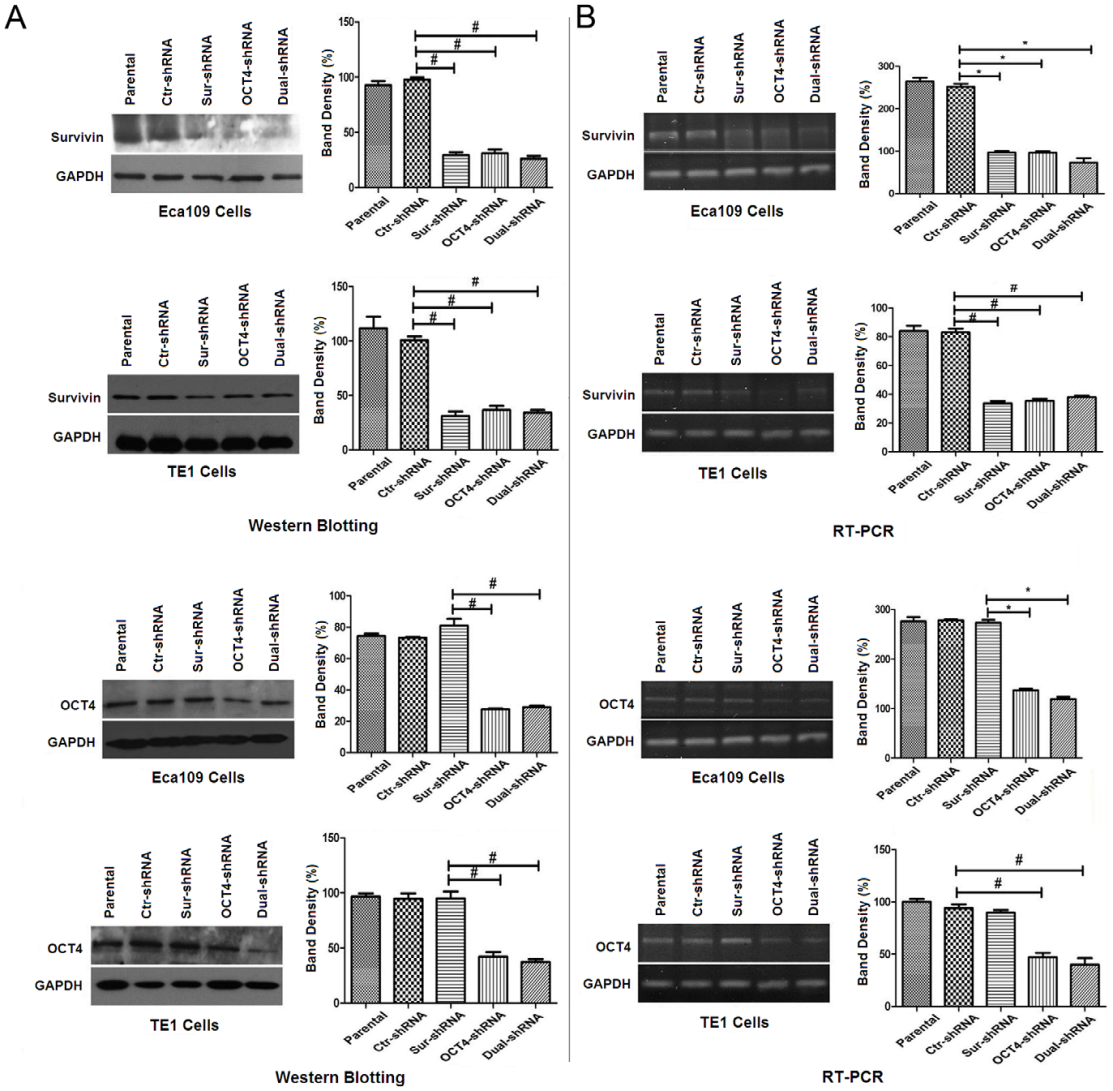
Survivin expression was associated with OCT4 in Eca109 and TE1 cells. (A) The parental and shRNA-transfected Eca109 and TE1 cells were cultured in 96-well plates at a density of 8×10^3^ cells/well for 48 h, and expression of OCT4 and Survivin in the harvested ESCC cells was examined by Western blotting. Glyceraldehyde-3- phosphatedehydrogenase (GAPDH) was used as a loading control. The relative band intensity was analyzed by densitometry after normalized with GAPDH contents, *p<0.05; ^#^ *p*<0.01. (B) OCT4 and Survivin mRNA expression were tested by reverse transcription polymerase chain reaction (RT-PCR). GAPDH was used as an inner control. The relative band intensity was analyzed by densitometry after normalizing with GAPDH contents, *p<0.05; ^#^ *p*<0.01. All data were representatives of 3 independent experiments. Quantitative histograms were pooled data. Error bars: standard deviation (SD); Dual-shRNA: vector containing double shRNAs targeting both OCT4 and Survivin.

To investigate the interaction and regulation between OCT4 and Survivin, the OCT4-shRNA, Sur-shRNA and Dual-shRNA vectors were designed to manipulate the target gene expression in ESCC cell lines. By Western blot and RT-PCR analysis, OCT4 and Survivin were positively expressed in the parental Eca109 and TE1 cells. The OCT4-shRNA and Sur-shRNA vectors could inhibit the specific target gene expression, and the Dual-shRNA vector could inhibit the expression of both OCT4 and Survivin genes. The OCT4-shRNA could also down-regulate the Survivin expression in Eca109 and TE1 cells (Fig. 4A), but the Sur-shRNA did not influence the OCT4 expression (Fig. 4B).

### Dynamic Localization of OCT4 and Survivin Expression in ESCC Cells

**Figure 5:**
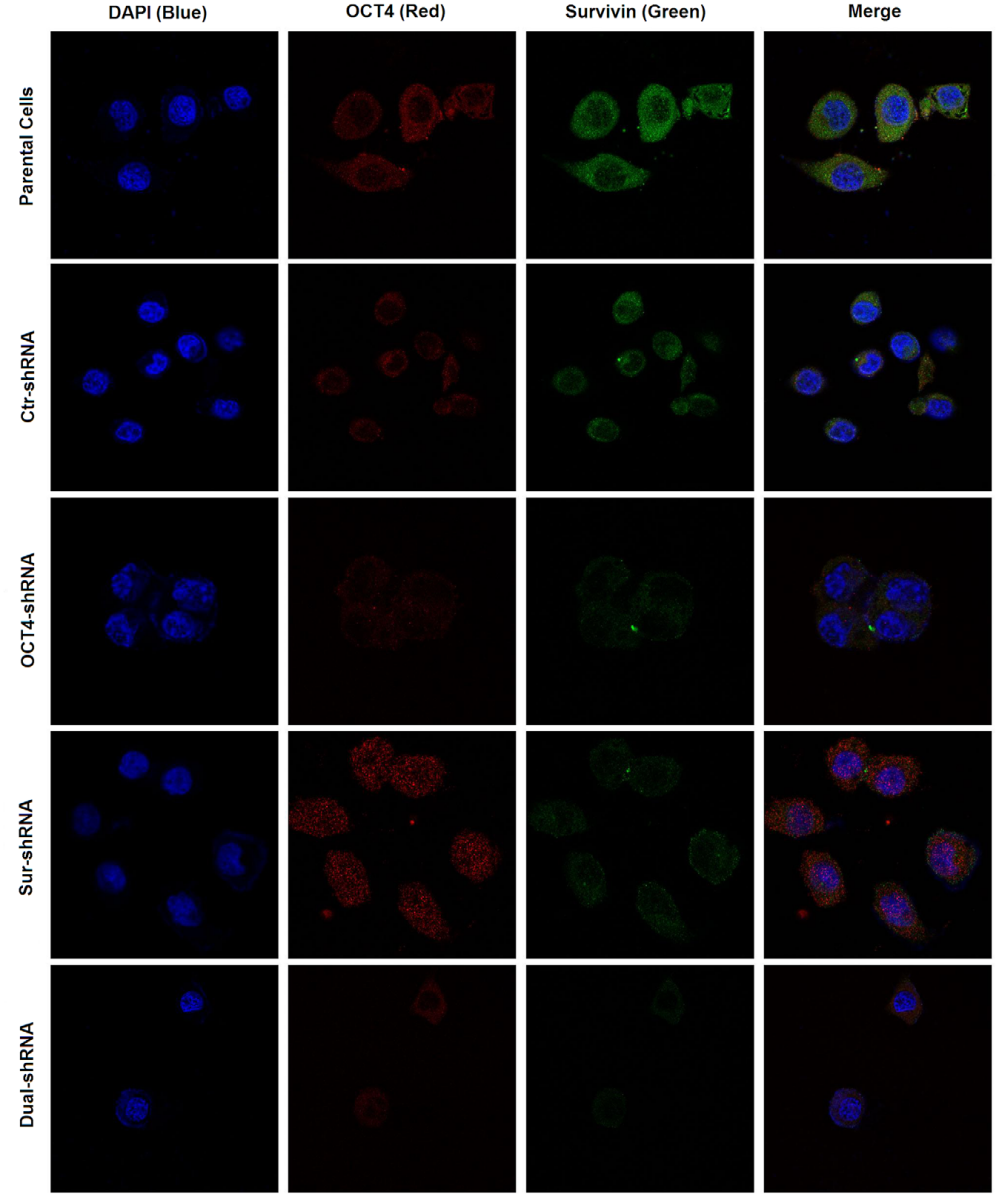
Subcellular localization of OCT4 and Survivin expression in ESCC cells. The parental and shRNA-transfected Eca109 and TE1 cells were cultured in 96-well plates at a density of 8×10^3^ cells/well for 48 h, and the harvested cells were fixed with 4% formaldehyde, then examined OCT4 and Survivin expression by immunoﬂuorescent confocal assay and observed under a laser-scanning confocal microscope.

We further investigated the dynamic variation of OCT4 and Survivin expression in ESCC cells by confocal assay. After 48 h transfection with OCT4-shRNA, Survivin expression was notably down-regulated in cancer cellular cytoplasm along with the decline of OCT4 expression in cellular nuclei. However, after transfection with Sur-shRNA, the OCT4 expression level was not altered along with the decrease of Survivin expression (Fig. 5).

## Discussion

OCT4, as one of the most important transcription factors, plays a pivotal role in pluripotent stem cells [26]. OCT4, together with SOX2 and Nanog, maintains stem cell pluripotency, self-renewal and differentiation [27]. It has gradually become a promising tumor biomarker for diagnosis of germ cell tumors [28]. Recently, it is reported that OCT4 could be detected in many somatic cell cancers from esophagus, bladder, lung, and liver, and has a strong influence on patients’ prognosis. The function of OCT4 could modulate a series of signal pathways, such as the Wnt/β-catenin, TGF-β, JAK/STAT3 signal pathways [29], [30], to activate or restrain the downstream target genes. Besides, OCT4 expression in mouse embryonic stem cells is necessary for protection from apoptosis and this effect may be associated with STAT3/Survivin pathway [25].

Survivin, a recently identified member of the inhibitors of apoptosis protein (IAPs) family, regulates the essential cellular process, including suppression of apoptosis, control of cell division, and promotion of angiogenesis [31]. As one of most prominent cancer genes, Survivin is expressed in almost all tumors, but is not detected in most normal adult tissue [32]. Survivin was considered as a target gene in cancer therapy, and down-regulation of Survivin could suppress tumor growth and improve tumor cell sensitivity to radiation and chemotherapy by promoting apoptosis and inhibiting cell viability. Drugs of LY2183108 [33] and YM155 [34] targeting Survivin were put into use in clinical trials in different stages and the effect was promising. Although tumor growth speed was slowed down on the strength of silencing Survivin, tumors still possess the ability of development and expansion and deprive of patients’ life, suggesting that Survivin was not a single factor for prognosis. There remains an unknown regulatory mechanism between OCT4 and Survivin.

Over-expression of OCT4 or Survivin in ESCC has been consistently connected with disease progression, metastatic dissemination, resistance to therapy [14], [18]. Therefore, we detected both OCT4 and Survivin expression in ESCC tumor specimens, and found that OCT4 and Survivin were closely related to the surgical outcome of ESCC patients. Patients with OCT4-positive or Survivin-positive tumors presented much poorer prognosis than those with OCT4-negative or Survivin-negative tumors. Among the subgroups, patients with OCT4-positive/Survivin-positive tumors showed the shortest overall survival time. By multivariate and univariate analyses, both OCT4 and Survivin are associated with patients’ prognosis, and OCT4 is considered as an independent factor for forcasting patients’ overall survival time. From our study, we concluded that OCT4 and Survivin were jointly related to the poor prognosis of ESCC patients, but the regulatory mechanisms between OCT4 and Survivin in ESCC are not yet clear.

Inhibiting the expression of OCT4 or Survivin in ESCC cell lines with the OCT4-shRNA or Sur-shRNA vectors resulted in a reduction in G2-phase cells and an increase in cell apoptosis, and co-suppression of OCT4 and Survivin by the Dual-shRNA vector resulted in an enhanced effect. Some studies showed that the inhibition of Survivin caused cell cycle arrest in G2-M phase [35], [36], but our study found the number of G2-phase cells was decreased tremendously after suppressing Survivin expression. G1-S phase, as well as G2-M phase, was the important checkpoint in cell cycle, which controls and maintains cell process accuracy. It was reported that the over-expression of Survivin can help cancer cells to pass G2-M checkpoint [37], [38]. At the same time, Survivin possesses a capability to upregulate the expression of Cyclin D1 and ensures cancer cells to pass G1-S checkpoint fluently and obtain an ability of persistent proliferation [38], [39].

Early apoptosis was increased with the downregulation of Survivin in many reports. To further investigate the molecular mechanism of OCT4-involved cell apoptosis and cell cycle arrest in Eca109 and TE1 cells, we found that the ESCC cells expressed lower levels of OCT4 and Survivin protein after transfected with OCT4-shRNA, but Sur-shRNA only down-regulated Survivin expression and there was no change in OCT4 expression level. Therefore, we concluded that OCT4 may regulate cell apoptosis and cell division through Survivin function. OCT4 could activate multiple signaling pathways to control Survivin expression, such as the STAT3, myc and NFκB pathways [30], [40]–[42], which is critical for cancer cell survival and antiapoptosis.

In the present study, we found that the over-expression of OCT4 and Survivin are an important feature in ESCC progression, and OCT4 expression is closely correlated with Survivin expression in the regulation of cancer cell apoptosis and proliferation. However, the molecular mechanisms between OCT4 and Survivin are very complicated. We should perform work to clarify the genetic features of OCT4 and Survivin, and design more efficient antitumor therapy for ESCC.

## Materials and Methods

### Patients and Samples

Fifty patients with ESCC were enrolled in the study in Changhai Hospital (Shanghai, China) from Jan 1 to Dec 31, 2005. The study was approved by the Ethic Committee of Second Military Medical University. The patients consisted of 37 men and 13 women. The age ranged from 47 to 72 years old, and the median age was 62 years old. All patients provided the informed written consent. The resected lesions were diagnosed pathologically as ESCC after surgery. The follow-up for patients started from the operation date to Aug 31, 2011. The overall survival (OS) was defined as the time period from the start date of follow-up to the date of death or the end date of follow-up.

### Cell Lines and Cell Culture

Human ESCC cell lines, Eca109 and TE1, were purchased from the Cell Bank of Chinese Academy of Sciences (Shanghai, China). Cells were maintained as a monolayer in RPMI-1640 with 10% FBS (fetal bovine serum), 100 IU/ml penicillin G and 100 µg/ml streptomycin, at 37°C in a humidified 5% CO_2_ incubator.

### Immunohistochemical Staining

The fresh tumors and adjacent normal tissues were obtained from the resected specimens during operation. The paraffin-embedded consecutive sections were subjected to immunohistochemical examination for OCT4 and Survivin expression using the primary antibodies, including mouse anti-human OCT4 (Santa Cruz Biotechnology, Inc. Santa Cruz, CA, USA) and rabbit anti-human Survivin (RD Systems Inc, Minneapolis, MN,USA), and the UltraSensitive Streptavidin Peroxidase Kit (Fuzhou Maixin Biotechnology Development Co., Fuzhou, China). The percentage of positive cells was counted within 5 high-power fields, and the evaluation of staining reaction was performed in accordance with the immunoreactive score standard proposed by Friedrichs [43].

### Construction and Transfection of shRNA Vectors

The shRNA sequences for human OCT4 (OCT4-shRNA: 5′-CCCTCACTTCACTGCACTG-3′) and Survivin (Sur-shRNA: 5′-GAAAGTGCGCCGTGCCATC-3′) were synthetized and cloned, respectively, into pGenesil vector (Wuhan Genesil Biotechnology Co., Ltd., Wuhan, China), in which the encoding sequences were controlled by the U6 promoter. The Dual-shRNA vector containing double shRNAs targeting both OCT4 and Survivin and mock control shRNA vector (Ctr-shRNA, 5′-GACTTCATAAGGCGCATGC-3′) were concomitantly constructed.

ESCC cells in log phase were transfected with shRNA vectors at a concentration of 4 µg/10^5^ cells using the Lipofectamine 2000 reagent (Invitrogen Corporation Shanghai Representative Office, Shanghai, China). The transfected cells were cultured continuously at 37°C in a CO_2_ incubator for another 48 h, then harvested for preparing to examine gene expression.

### Western Blotting Analysis

The harvested cells were lysed in phenylmethanesulfonyl fluoride (PMSF) solution, and the isolated protein was electrophoresed on sodium dodecyl sulfate polyacrylamide gel electrophoresis (SDS-PAGE) and examined by Western blotting using the anti-Survivin and anti-OCT4 antibodies. Blots were visualled by the enhanced chemiluminescence reagent (PerkinElmer Inc., Shanghai, China).

### Semiquantitative Reverse Transcription Polymerase Chain Reaction (RT-PCR)

After 48 h of transfection, total RNA was extracted with Trizol reagent (Invitrogen, Carlsbad, California, USA) from the harvested Eca109 and TE1 cell lines, and dissolved in diethylpyrocarbonatetreated (DEPC) water. The expression of OCT4 and Survivin was examined using the Takara Reverse Transcription System Kit (Takara Biotechnology Co. Ltd., Dalian, China) by the OCT4 primers (sense: 5′-CAG TCGTCAGCGTCGTCGTTGTAAGCTGCGGCCC-3′, antisense: 5′-ACGCGTCG ACTCAGTTTGAATGCATGGGAG-3′, products 480-bp) and the Survivin primers (sense: 5′-CGGAATTCACCATGGGTGCCCCGACG-3′, antisense: 5′-GAAGATC TTCAATCCATGGCAGCCAG-3′, products 448-bp). Glyceraldehyde-3- phosphatedehydrogenase (GAPDH) was used as a loading control by the primers (sense: 5′-ACCACAGTCCATGCCATCAC-3′, antisense: 5′-TCCACCACCCTGT TGCTTGTA-3′, products 120-bp). Bands of the products electrophoresed on ethidium bromide (EB)-agarose gel were analyzed semiquantitatively by grayscale densitometry analysis.

### Analysis of Cell Cycle and Apoptosis by Flow Cytometry (FCM)

After 48 h of transfection, the harvested cells washed by 0.1 M phosphate buffered saline (PBS) solution and resuspended cells with 0.1 M PBS to the concentration of 10^6^ cells/ml. A part of cells were fixed in 1 ml pre-cooled 70% alcohol overnight at 4°C followed by incubation with propidium iodide (PI) solution at the concentration of 50 µg/ml and RNase A at the concentration of 20 µg/ml for 30 min in darkness. The cell cycle was analyzed by FCM (FACS420, BD Biosciences, San Jose, CA). Another part of cells were incubated with 5 µl Annexin V-FITC in 195 µl binding buffer in darkness for 10 min. Cells were centrifuged at 1000 g for 5 min and resuspended in 190 µl binding buffer and 10 µl PI, followed by ﬂuorescence analysis.

### Methylthiazoletetrazolium Assay

Eca109 and TE1 cells were seeded in 96-well plates at a density of 8,000 cells/well and transfected with shRNA vector as described above. After 48 h, 3-(4, 5)-dimethylthiahiazo(-z-y1)-3,5-diphenytetrazoliumromide (MTT) at 5 mg/ml was added into each well and continuously incubated for 4 h. After added 150 µl dimethyl sulfoxide (DMSO) to each well, the absorbance was measured at 490 nm.

### Immunoﬂuorescent Confocal Assay

Cancer cells were harvested and fixed with 4% formaldehyde for 15 min at room temperature, then washed in 0.1 M PBS and treated with 0.3% Triton X-100, followed by incubation with the indicated OCT4 (1∶100) and Survivin (1∶100) primary antibodies at 4°C overnight and the secondary antibodies, ﬂuorescein isothiocyanate (FITC)-conjugated anti-rabbit IgG or cyanine-3 (Cy3)-labeled goat anti-mouse IgG (Santa Cruz Biotechnology, Inc., Santa Cruz, CA), at room temperature for 40 min in darkness. Cells were stained by 4′,6-diamidino-2-phenylindole (DAPI) and detected under a laser-scanning confocal microscope.

### Statistical Analysis

The chi-square test and Spearman’s rank correlation were used to analyze the relationship and correlation between relative gene expression and clinical pathological data. OS was assessed by the Kaplan–Meier method and the significant difference in OS was calculated by log-rank test. Univariate and multivariate analyses were performed using Cox’s proportional hazard model. The experimental data were representative of three independent experiments, and analyzed statistically by two-way analysis of variance (ANOVA) using the PASW Statistics software version 18.0 (SPSS, Chicago, Illinois, USA). Differences were considered to be significant for *p*<0.05.
